# A preconsultation web-based tool to generate an agenda for discussion in diabetes outpatient clinics to improve patient outcomes (DIAT): a feasibility study

**DOI:** 10.1136/bmjopen-2016-013519

**Published:** 2017-03-07

**Authors:** Obioha C Ukoumunne, Bijay Vaidya, Julia Frost, Rob Anderson, Catherine Argyle, Mark Daly, Faith Harris-Golesworthy, Jim Harris, Andy Gibson, Wendy Ingram, Jon Pinkney, Jane Vickery, Nicky Britten

**Affiliations:** 1 NIHR CLAHRC South West Peninsula (PenCLAHRC), University of Exeter Medical School, Exeter, UK; 2 Department of Diabetes & Endocrinology, Royal Devon and Exeter Hospital, University of Exeter Medical School, Exeter, UK; 3 Institute of Health Research, University of Exeter Medical School, Exeter, UK; 4 Peninsula Technology Assessment Group (PenTAG), Institute of Health Research, University of Exeter Medical School, Exeter, UK; 5 Macleod Diabetes and Endocrine Centre, Royal Devon and Exeter Foundation Trust, Exeter, UK; 6 Peninsula Public Involvement Group (PenPIG), NIHR CLAHRC South West Peninsula (PenCLAHRC), University of Exeter Medical School, Exeter, UK; 7 Peninsula Clinical Trials Unit, Plymouth University Peninsula Schools of Medicine and Dentistry, Plymouth, UK; 8 Plymouth University and Peninsula Schools of Medicine and Dentistry, Derriford Hospital, Plymouth Hospitals NHS Trust, Plymouth, UK

**Keywords:** diabetes, patient enablement, self-management, pre-consultation

## Abstract

**Objective:**

To test the feasibility of running a randomised controlled trial of a preconsultation web-based intervention (Presenting Asking Checking Expressing (PACE-D)) to improve the quality of care and clinical outcomes in patients with diabetes.

**Design and setting:**

A feasibility study (with randomisation) conducted at outpatient diabetes clinics at two secondary care hospitals in Devon, UK.

**Participants:**

People with diabetes (type 1 and type 2) attending secondary care general diabetes outpatient clinics.

**Intervention:**

The PACE-D, a web-based tool adapted for patients with diabetes to use before their consultation to generate an agenda of topics to discuss with their diabetologist.

**Outcomes:**

The percentage of eligible patients who were recruited and the percentage of participants for whom routine glycosylated haemoglobin (HbA1c) data (the putative primary outcome) could be extracted from medical notes and who completed secondary outcome assessments via questionnaire at follow-up were reported.

**Results:**

In contrast with the planned recruitment of 120 participants, only 71 participants were randomised during the 7-month recruitment period. This comprised 18.7% (95% CI 14.9% to 23.0%) of those who were eligible. Mean (SD) age of the participants was 56.5 (12.4) years and 66.2% had type 1 diabetes. Thirty-eight patients were randomised to the intervention arm and 33 to the control arm. HbA1c data were available for only 73% (95% CI 61% to 83%) of participants at the 6 months follow-up. The questionnaire-based data were collected for 66% (95% CI 54% to 77%) of the participants at 6 months follow-up. Participants reported that the PACE-D tool was easy to use.

**Conclusions:**

A randomised controlled trial of the preconsultation web-based intervention as set out in our current protocol is not feasible without significant modification to improve recruitment and follow-up of participants. The study also provides insights into the feasibility and challenges of conducting complex intervention trials in everyday clinical practice.

**Trial registration:**

ISRCTN75070242.

**Strengths and limitations of the study**

- Patients are involved in the development of the intervention.
- The trial is conducted at two hospital sites.
- Outcomes are comprised of self-reported patient data collected via questionnaire and routine glycosylated haemoglobin measurements.
- The patients, clinicians and healthcare staff are not blinded to intervention status.

## Introduction

Diabetes is a common chronic condition affecting about 4 million people in the UK and by 2025 it is estimated that the prevalence will rise to over 5 million.^1^ People with diabetes are at an increased risk of developing several serious long-term complications, such as ischaemic heart disease, stroke, chronic kidney disease, blindness and amputations, associated with disability and premature death as well as a huge economic cost to the individual and society. Optimal control of diabetes can prevent or delay the onset of these complications.^2^ ^3^

Although diabetes is largely a self-managed condition, specialist healthcare professionals have a vital role to play by helping patients to achieve good diabetes control and to cope with their illness through expert advice, education and support.^4^ Effective consultations with healthcare professionals have been shown to enhance patient empowerment, promote positive behaviour change and improve diabetes outcomes.^5–7^ However, consultations with healthcare professionals tend to be infrequent and time limited, and patients often feel unable to discuss their concerns.^8^ Therefore, interventions that enable patients to discuss their concerns may be advantageous in encouraging better self-management.

Cegala and colleagues^9^ have previously suggested that communication skills training for patients can enhance their participation in the medical consultation. They have proposed the Presenting Asking Checking Expressing (PACE) system for patients to develop effective communication; this involves patients *presenting* detailed information about how they are feeling, *asking* questions if desired information is not provided, *checking* understanding of information that is given to them and *expressing* any concerns about the recommended treatment.^10^ The PACE system was modified specifically for diabetes to produce a web-based tool (designated PACE-diabetes or PACE-D), designed to be completed by a patient immediately before a clinic appointment with a diabetes specialist to identify the issues that they would wish to discuss in the clinic (ie, their ‘agenda’). Web-based educational interventions have recently been evaluated for patients with diabetes.^11^ ^12^

Here, we report results of a randomised controlled trial that we carried out to assess the feasibility of and obtain the necessary information for planning a future definitive trial of this web-based preconsultation intervention (PACE-D) to improve the care quality and clinical outcomes of patients with diabetes. Findings from qualitative work carried out alongside the trial (with the aims of exploring patient and public involvement (PPI) in trial processes, patient experience and relevant organisational factors) are reported in separate papers.

## Setting, subjects and methods

This feasibility study used a pragmatic parallel group randomised controlled trial design (trial registration number: ISRCTN75070242) with the overall aim of establishing the feasibility of a definitive superiority trial of the PACE-D agenda setting tool.^13^

### Study setting and recruitment

Patients with diabetes were recruited from two sites (Macleod Diabetes and Endocrine Centre at the Royal Devon & Exeter Hospital, Exeter and the Medical Outpatients Department at Derriford Hospital, Plymouth) in Devon, South West England. Eligible patients met all of the following criteria: aged 18 years or over; with type 1 or type 2 diabetes mellitus; due to attend for a hospital outpatient appointment with a diabetologist and had sufficient written and spoken English to complete the study assessments. Women with gestational diabetes and patients receiving insulin pump therapy were excluded as these patients are seen in specialised clinics.

Potentially eligible patients who were due to attend a general diabetes clinic appointment were identified from clinic lists by healthcare assistants who had been specially trained for the study. Patients who were willing to be contacted were sent an information sheet about the trial and were contacted by phone by the healthcare assistant no less than a week later to confirm eligibility and discuss the study. Patients who wanted to take part in the study were then sent a consent form and baseline questionnaire. On receipt of the signed consent form, the patient was randomised.

### Randomisation

Participants were randomised in a 1:1 ratio to the intervention or control arms using a computer-generated random allocation sequence prepared by the Peninsula Clinical Trials Unit at Plymouth University (PenCTU). An automated web-based system was used to conceal the allocation. Randomisation was stratified by clinic session using randomly permuted block sizes in a non-systematic sequence. Allocation occurred on receipt of the patient's completed consent form. Following allocation, the hospital informed the patient, via a standard letter, of the time of their clinic appointment. Participants in the intervention arm were asked to attend 30 min before the start of their appointment to ensure sufficient time to complete the PACE-D tool. For both trial arms, if the baseline questionnaire had not been returned to the CTU before the clinic appointment, the participant was asked to complete it in clinic, prior to the consultation (and prior to the PACE-D for those in the intervention arm).

### Interventions

The PACE-D intervention is a web-based tool used by the patient before their consultation to generate an agenda of topics to discuss with their diabetologist. It is based on the PACE tool developed by Cegala and colleagues^9^ and was modified for use with patients with diabetes by the DIAT study team and PPI representatives (ie, people with experience of living with diabetes). A review of the literature had identified several candidate interventions, which were discussed and tested by the study team. PACE had been shown to enable patient communication skills to move beyond noting concerns, allowing the patient to take an active role in shaping the dynamic of the physician–patient relationship and the flow of information.^14^ The PACE curricula were tailored to diabetes specifically, with input from the two PPI coauthors who shaped the development of the intervention with the web designers at PenCTU, using easy-to-access information from the Diabetes UK website (https://www.diabetes.org.uk/Guide-to-diabetes/?gclid=CITP94CthdACFQEA0wodVjMJBQ) and their experiential knowledge. A working group of eight people with diabetes was convened by the PPI facilitator. In a 3-hour workshop, this group tested and provided feedback on this early iteration of the intervention. The intervention was fine-tuned using an iterative process.

The PACE-D consists of several open and closed questions, prompts and a list of potential concerns the patient might have (eg, depression). A healthcare assistant, trained to facilitate the intervention, was on hand to provide as much help to the patients as required, but without influencing their choices, so that they could go through the PACE-D questions and identify the problems and topics that they wished to discuss in their consultation. After completion of the PACE-D (which takes ∼20 min), a printed personalised consultation agenda was generated for the patient to take into the consultation. The following documents are included as online supplementary material with this paper: the text used in the PACE intervention; the offline version of the PACE-D intervention and an example of an agenda that was generated for a study patient using the PACE-D.

10.1136/bmjopen-2016-013519.supp1 supplementary material

Patients randomised to the control arm received their standard outpatient appointment with a diabetologist.

### Data collection/measures

Data were collected at baseline, 3 and 6 months comprising results of routine measurement of glycaemic control extracted from patient records and several patient-reported measures sent to participants with instructions for completion and a prepaid return envelope. We briefly describe the patient-reported measures. Further details are presented in the protocol paper.^13^

Glycaemic control, the putative primary outcome for the definitive trial of the PACE-D tool, was quantified using *glycosylated haemoglobin* (HbA1c) retrospectively obtained from participants' medical records. These data were therefore obtained opportunistically from routine measurements the timing of which was unconnected to the aims of the study. To be used in this study at a given wave, the HbA1c level needed to be measured within 4 weeks of the intended assessment date.

The *Audit of Diabetes-Dependent Quality of Life-19 (ADDQoL)* measures the patient's perception of the impact of diabetes on their quality of life weighted by its importance to the individual.^15^ The mean impact score (possible scoring range from −9 to +3) across 19 domains (eg, working life, holidays, physical appearance, etc) was analysed and reported here. Higher scores indicate a more positive state.

The *Diabetes Empowerment Scale*-short form (DES) measures diabetes-related psychosocial self-efficacy.^16^ ^17^ An overall score for DES (possible range 1–5) is calculated by taking the mean of the eight constituent items, with higher scores indicating greater self-efficacy.

The *Diabetes Self-care Activity* self-report questionnaire is a measure of diabetes self-management which includes five aspects (activities) of the diabetes regimen: 18 general diet, specific diet, exercise, blood glucose testing and foot care. The overall score (possible scoring range 0–7) for each activity is based on the number of days in the past week that the activity was undertaken.

The *Diabetes Treatment Satisfaction Questionnaire—Status* (DTSQ(S)) measure and change (DTSQ(C)) versions were developed to measure patient satisfaction with diabetes treatment.^19^ The total score for the DTSQ(S) ranges from a possible 0–36 with higher scores indicating greater satisfaction. The DTSQ change version (DTSQ(C)) contains the same items, but asks patients to consider their satisfaction with current treatment compared with their previous treatment.^20^ The total score ranges from a possible score −18 to 18. Both versions of the DTSQ were used in order to capture initial perceptions and any change at follow-up.

The *Patient Enablement Instrument* (PEI) measures patient enablement after a consultation with a physician.^21^ The total score ranges from 0 to 12. We used the modified version of Haughney *et al*,^22^ in which the opening statement captures perspectives on treatment specifically. Higher scores indicate greater enablement.

*Patient Report of Communication*. Developed to measure communication in conjunction with the PACE tool; this instrument comprises 11 questions about perceived communication, with two items for each of the four PACE skills, two additional items for the patient's ability to state their preferences and a global item about the consultation.^14^ It uses a 5-point Likert scale format that captures aspects of doctor–patient communication as never (1), not very often (2), sometimes (3), usually (4) and always (5). The total score was calculated as the sum over the individual items with higher scores indicating better communication.

The *EuroQoL* (EQ-5D-5L) was used to measure generic health-related quality of life. It defines health in terms of: mobility, self-care, usual activities, pain/discomfort and anxiety/depression and uses a 3-point Likert scale (no problem, some problem, extreme problem).^23^ However, this instrument has also been found to suffer from ceiling effects, and a five-level version has been developed (EQ-5D-5L), which uses a five-point Likert scale (no problem, slight problem, moderate problem, severe problem and extreme problem) and has demonstrated increased reliability and sensitivity.^24^

The *Client Services Receipt Inventory* was used to collect cost-related and service use-related information for trial participants.^25^ However, as this paper focuses primarily on trial feasibility and assessment of clinical outcomes, we do not report the service use and cost data in this paper (but they are available from the authors).

*Other outcomes*. Participants were asked to document their current medication at each time point, as well as the number and type of contacts with primary and secondary care practitioners during the study. Finally, those in the intervention arm were also asked at 6 months about their experience of using the PACE-D tool and the utility of producing their agenda.

### Blinding

It was not possible to blind the participating patients, the consulting diabetologists or the healthcare assistants due to the nature of the intervention which results in the generation of a consultation agenda.

### Sample size

The study aimed to recruit 120 patients in total. This is large enough to estimate the percentage that is lost to follow-up at 3 and 6 months with a margin of error no greater than ±9.3% based on the 95% CI width. Assuming that at least 60 patients provide follow-up data, this is large enough to estimate the SD of the continuous outcome measures to within 22% of their true value based on the upper limit of the 95% CI.

### Statistical analysis

The main objectives of this trial were to affirm aspects of the feasibility of a definitive trial and estimate parameters for planning recruitment and calculating the sample size for such a study. The percentage of eligible patients who were recruited, the percentage of participants for whom HbA1c data could be extracted from medical notes and the percentage of participants that completed outcome assessments at follow-up are reported with 95% CIs.

Characteristics of study participants (by trial arm status) and eligible non-participants are summarised using means and SDs for continuous variables and numbers and percentages for categorical variables. The SDs of the outcome measures (all continuous) are reported with 95% CIs for each trial arm at each of the 3 and 6 month follow-ups.

In ancillary analyses, we used the intention-to-treat principle to compare trial arms with respect to the study outcomes (all continuous) using the t-test for crude estimates of the mean difference and linear regression for estimates adjusted for study site, time since diagnosis and the baseline score for the measure. Ninety-five per cent CIs for the adjusted mean difference are reported, but no p values as this is a feasibility study.

## Results

**Figure 1:**
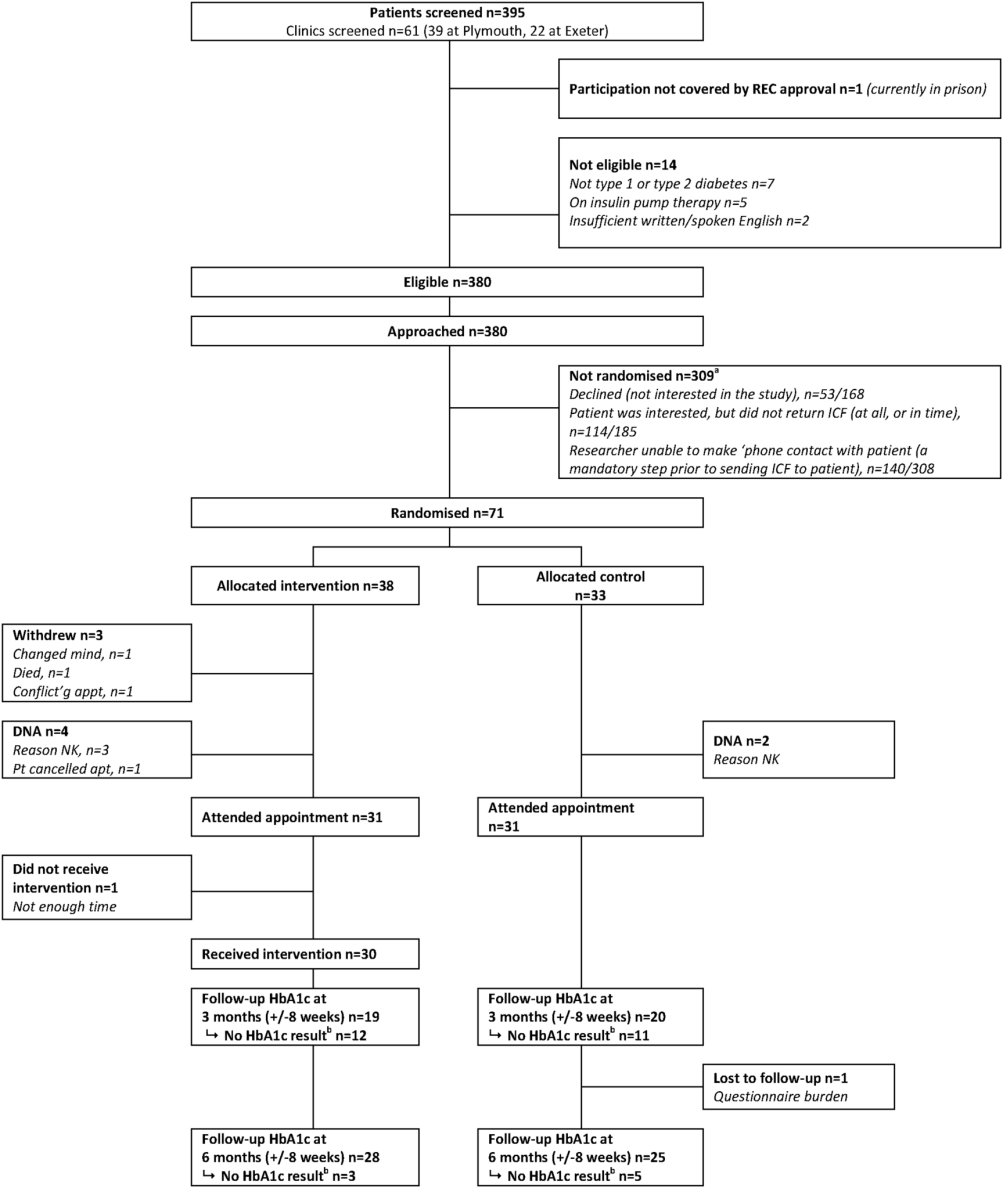
CONSORT diagram. ^a^The recruitment process was amended during the recruitment phase, and the denominator for the reasons that patients were not randomised varies accordingly. ^b^There was no HbA1c result within the time window recorded on the participating hospital's laboratory database.

**Figure 2:**
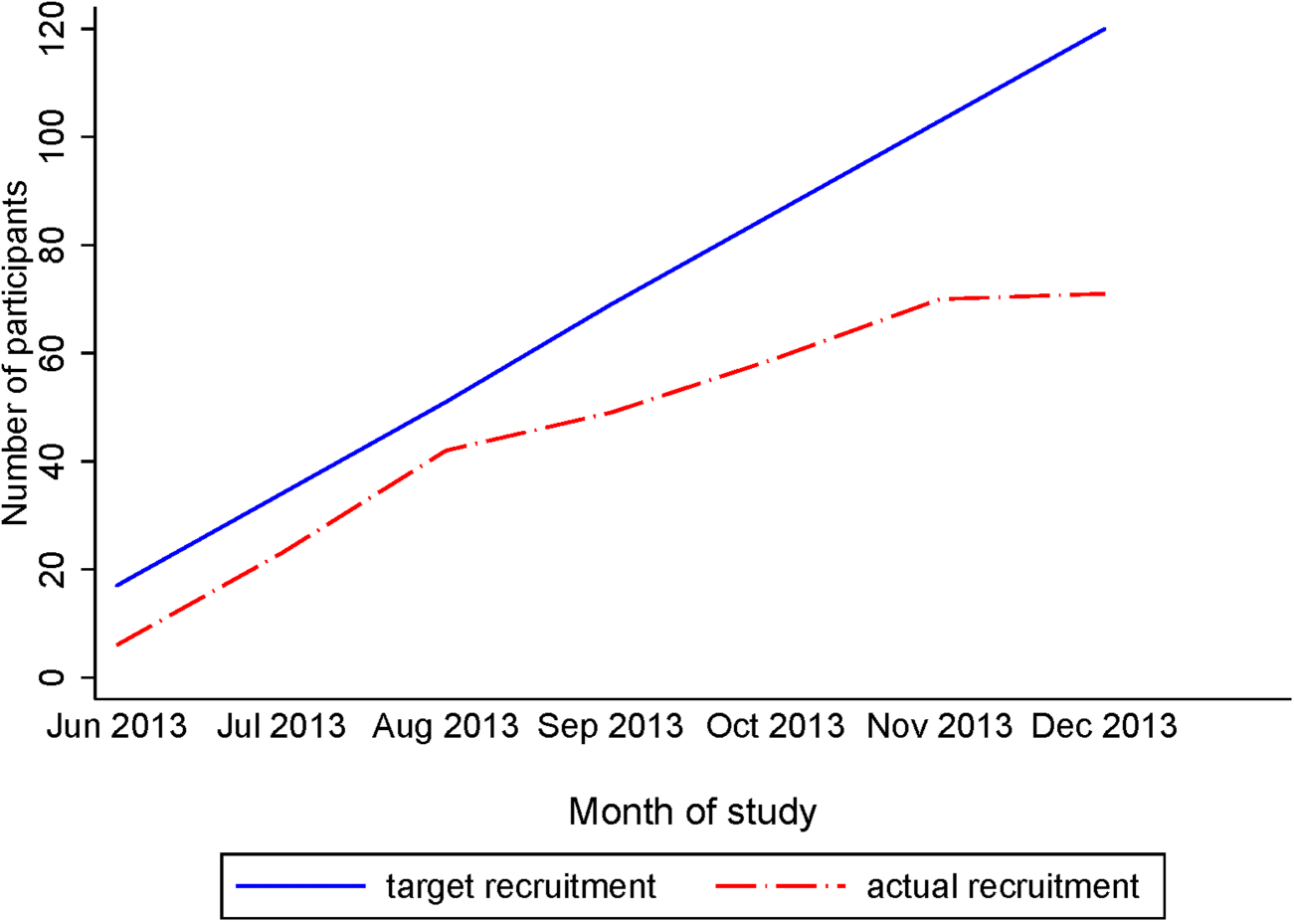
Final recruitment graph (24 June 2013 to 31 December 2013).

The participant flow through the trial is summarised in figure 1. Recruitment took place between 24 June 2013 and 31 December 2013. As shown in figure 2, the recruitment was slow to start and generally lower than anticipated particularly in the latter months. Efforts were made to streamline the recruitment process including the following protocol changes: adding an option for interested potential participants to contact the screening team directly rather than awaiting a call; getting a healthcare assistant rather than a research nurse to check if a participant is still willing to participate when they attend the outpatient appointment and reducing the time between sending the initial invitation letter and the telephone call made to confirm eligibility and discuss the study.

Three hundred and ninety-five patients with diabetes on the waiting list for appointment in the general diabetes outpatients clinics were screened for eligibility by reviewing their case records; 380 of these were eligible for the trial of whom 71 were recruited, giving a participation percentage of 18.0% (95% CI 14.3% to 22.1%) out of those who were screened and 18.7% (95% CI 14.9% to 23.0%) out of those who were eligible. The number recruited was 59 patients fewer than targeted. Participation was slightly higher in Exeter than Plymouth (24.8% of those who were eligible vs 15.8%). Participants were similar to eligible non-participants with respect to mean (SD) age at registration (56.5 (12.4) versus 51.3 (16.1)), type 1 (as opposed to type 2) diabetes status (66.2% vs 60.5%) and whether attending for a new appointment (4.2% vs 8.1%).

Thirty-eight participants were randomised to the intervention arm and 33 to the control arm; the baseline characteristics are summarised by trial arm status in table 1. The 3-month follow-up questionnaires were received between 25 September 2013 and 8 May 2014 and the 6-month follow-up questionnaires between 23 December 2013 and 15 August 2014. The number of participants for whom HbA1c data could be extracted and who provided questionnaire data at each wave is reported in table 2. There was a large amount of missing data on HbA1c with only 73% (95% CI 61 to 83%) having a routine assessment within 4 weeks of the scheduled 6-month follow-up date. Even fewer (66%; 95% CI 54 to 77%) provided questionnaire data at that wave.

**Table 1:** Baseline characteristics by trial arm status

Characteristics	Intervention N=38	Control N=33
Female patient, %	50	45
Age at registration, mean (SD)	55.3 (12.4)	57.8 (12.6)
White, %	96	93
Height in cm, mean (SD)	167 (7)	171 (8)
Weight in kg, mean (SD)	95 (27)	93 (27)
Time since diagnosis		
<5 years, %	19	15
5–10 years, %	23	8
10–20 years, %	27	42
20 years or more, %	31	35
Diagnosed by		
GP, %	69	76
Consultant, %	31	24
Diabetes status		
Type I, %	61	73
Type II, %	39	27
HbA1c, mean (SD)	70.7 (17.1)	68.2 (14.9)
ADDQoL—average weighted impact score, mean (SD)	−1.6 (1.5)	−2.8 (2.2)
Diabetes Empowerment Scale—short form, mean (SD)	3.4 (0.6)	3.6 (0.6)
DSCAQ—General Diet score, mean (SD)	4.7 (2.1)	4.5 (2.3)
DSCAQ—Specific Diet score, mean (SD)	4.2 (1.6)	4.4 (1.9)
DSCAQ—Exercise score, mean (SD)	3.3 (2.4)	3.0 (2.7)
DSCAQ—Blood Glucose Testing score, mean (SD)	4.9 (2.4)	5.9 (1.9)
DSCAQ—Foot Care score, mean (SD)	2.9 (2.2)	2.8 (2.4)
Diabetes Treatment Satisfaction Questionnaire score, mean (SD)	27.7 (6.8)	28.7 (6.0)
Patient Enablement Instrument, mean (SD)	8.4 (4.0)	7.2 (3.8)
Patient Report of Communication Behaviour, mean (SD)	38.7 (8.0)	39.3 (8.7)

Sample size ranges from 23 to 38 in the intervention arm and from 24 to 33 in the control arm.

ADDQoL, Audit of Diabetes-Dependent Quality of Life; GP, general practitioner; HbA1c, glycosylated haemoglobin.

**Table 2:** Three and 6-month outcome results by trial arm

Outcome and time point	Intervention		Control		Intervention—control	
	N	mean (SD)	N	mean (SD)	mean diff.	adj. mean diff. (95% CI)
3 months						
HbA1c	16	67.4 (17.6)	16	66.6 (10.4)	0.8	0.4 (−11.9 to 12.7)
ADDQoL—average weighted impact score	25	−1.4 (1.4)	26	−2.6 (2.0)	1.2	0.3 (−0.6 to 1.1)
Diabetes Empowerment Scale—short form	22	3.6 (0.5)	25	3.6 (0.7)	0.03	0.01 (−0.4 to 0.4)
DSCAQ—General Diet score	24	4.7 (2.3)	24	4.5 (2.3)	0.2	0.05 (−1.1 to 1.2)
DSCAQ—Specific Diet score	25	3.9 (2.1)	25	4.1 (1.6)	−0.2	0.008 (−0.9 to 0.9)
DSCAQ—Exercise score	25	3.2 (2.2)	24	2.8 (2.4)	0.4	−0.2 (−1.3 to 1.0)
DSCAQ—Blood Glucose Testing score	23	5.2 (2.0)	24	6.1 (1.7)	−0.8	−0.03 (−1.2 to 1.2)
DSCAQ—Foot Care score	25	3.6 (2.3)	24	3.3 (2.4)	0.3	0.2 (−0.8 to 1.1)
Diabetes Treatment Satisfaction Questionnaire score	24	28.4 (6.0)	25	28.6 (6.3)	−0.2	0.7 (−2.8 to 4.2)
Diabetes Treatment Satisfaction Questionnaire change score	24	4.8 (9.9)	22	4.4 (8.3)	0.4	0.1 (−6.8 to 7.0)
Patient Enablement Instrument	23	8.1 (3.6)	24	7.5 (4.1)	0.6	−0.5 (−2.6 to 1.6)
Patient Report of Communication Behaviour	24	42.9 (6.0)	25	39.3 (6.8)	3.6	2.1 (−1.1 to 5.2)
6 months						
HbA1c	26	71.6 (16.8)	26	71.1 (20.4)	0.5	−3.4 (−13.1 to 6.3)
ADDQoL—average weighted impact score	23	−1.4 (1.6)	24	−2.2 (1.9)	0.8	−0.1 (−1.0 to 0.7)
Diabetes Empowerment Scale—short form	23	3.7 (0.5)	24	3.7 (0.6)	−0.04	−0.008 (−0.4 to 0.4)
DSCAQ—General Diet score	23	4.5 (2.0)	24	5.1 (2.4)	−0.6	−0.9 (−2.1 to 0.3)
DSCAQ—Specific Diet score	23	3.8 (1.2)	24	4.4 (1.2)	−0.7	−0.3 (−1.0 to 0.3)
DSCAQ—Exercise score	23	3.5 (2.3)	20	2.4 (2.4)	1.1	1.4 (0.09 to 2.6)
DSCAQ—Blood Glucose Testing score	23	5.2 (2.3)	23	6.4 (1.4)	−1.2	0.08 (−1.1 to 1.2)
DSCAQ—Foot Care score	23	3.7 (2.2)	23	3.3 (2.7)	0.4	0.4 (−0.9 to 1.6)
Diabetes Treatment Satisfaction Questionnaire score	22	30 (7.2)	21	29.0 (6.4)	1.0	0.7 (−3.5 to 5.0)
Diabetes Treatment Satisfaction Questionnaire change score	21	6.6 (11.1)	22	4.4 (11.9)	2.2	0.9 (−7.3 to 9.2)
Patient Enablement Instrument	19	9.8 (3.3)	22	7.2 (3.9)	2.6	2.5 (0.8 to 4.1)
Patient Report of Communication Behaviour	23	42.1 (7.3)	24	41.7 (7.1)	0.4	2.0 (−3.0 to 7.1)

ADDQoL, Audit of Diabetes-Dependent Quality of Life; HbA1c, glycosylated haemoglobin.

Table 2 summarises the comparison of the study outcomes between the intervention and control arms at 3 and 6 months. These are ancillary analyses, as the primary aim of this feasibility study is to estimate the parameters required for planning a definitive trial. Reflecting the small sample size, the CIs for the differences between the trial arms were too wide to be confident of a definite benefit or harm related to the intervention or to rule out the possibility of benefits or harms. Table 3 reports the SD of the outcomes at each of the 3 and 6 month follow-ups by trial arm status with 95% CIs. Table 4 summarises the responses to the *EuroQoL* items.

**Table 3:** SD (95% CI) of outcome measures at 3 and 6 month follow-ups by trial arm status

Measure	3 month follow-up		6 month follow-up	
	Intervention	Control	Intervention	Control
HbA1c	17.6 (13.0 to 27.2)	10.4 (7.7 to 16.1)	16.8 (13.2 to 23.2)	20.4 (16.0 to 28.2)
ADDQoL—average weighted impact score	1.4 (1.1 to 1.9)	2.0 (1.6 to 2.8)	1.6 (1.2 to 2.3)	1.9 (1.5 to 2.7)
Diabetes Empowerment Scale—short form	0.5 (0.3 to 0.7)	0.7 (0.5 to 1.0)	0.5 (0.4 to 0.7)	0.6 (0.5 to 0.8)
DSCAQ—General Diet score	2.3 (1.8 to 3.2)	2.3 (1.8 to 3.2)	2.0 (1.6 to 2.8)	2.4 (1.9 to 3.4)
DSCAQ—Specific Diet score	2.1 (1.6 to 2.9)	1.6 (1.2 to 2.2)	1.2 (0.9 to 1.7)	1.2 (0.9 to 1.7)
DSCAQ—Exercise score	2.2 (1.7 to 3.1)	2.4 (1.9 to 3.4)	2.3 (1.8 to 3.3)	2.4 (1.8 to 3.5)
DSCAQ—Blood Glucose Testing score	2.0 (1.5 to 2.8)	1.7 (1.3 to 2.4)	2.3 (1.8 to 3.3)	1.4 (1.1 to 2.0)
DSCAQ—Foot Care score	2.3 (1.8 to 3.2)	2.4 (1.9 to 3.4)	2.2 (1.7 to 3.1)	2.7 (2.1 to 3.8)
Diabetes Treatment Satisfaction Questionnaire score	6.0 (4.7 to 8.4)	6.3 (4.9 to 8.8)	7.2 (5.5 to 10.3)	6.4 (4.9 to 9.2)
Diabetes Treatment Satisfaction Questionnaire change score	9.9 (7.7 to 13.9)	8.3 (6.4 to 11.9)	11.1 (8.5 to 16.0)	11.9 (9.2 to 17.0)
Patient Enablement Instrument	3.6 (2.8 to 5.1)	4.1 (3.2 to 5.8)	3.3 (2.5 to 4.9)	3.9 (3.0 to 5.6)
Patient Report of Communication Behaviour	6.0 (4.7 to 8.4)	6.8 (5.3 to 9.5)	7.3 (5.6 to 10.3)	7.1 (5.5 to 10.0)

ADDQoL, Audit of Diabetes-Dependent Quality of Life; HbA1c, glycosylated haemoglobin.

**Table 4:** Responses to EuroQol items at 3 and 6 months by trial arm

EuroQol item	3 months		6 months	
	Intervention	Control	Intervention	Control
	n (%)	n (%)	n (%)	n (%)
Mobility				
No problems	15 (60)	10 (40)	13 (57)	11 (46)
Slight problems	5 (20)	2 (8)	4 (17)	3 (13)
Moderate problems	4 (16)	9 (36)	4 (17)	6 (25)
Severe problems	1 (4)	2 (8)	2 (9)	3 (13)
Unable to/extreme	0 (0)	2 (8)	0 (0)	1 (4)
Self-care				
No problems	21 (84)	13 (50)	19 (83)	14 (58)
Slight problems	2 (8)	6 (23)	1 (4)	3 (13)
Moderate problems	1 (4)	5 (19)	3 (13)	5 (21)
Severe problems	1 (4)	1 (4)	0 (0)	1 (4)
Unable to/extreme	0 (0)	1 (4)	0 (0)	1 (4)
Usual activities				
No problems	18 (72)	11 (42)	12 (55)	11 (46)
Slight problems	2 (8)	5 (19)	4 (18)	5 (21)
Moderate problems	3 (12)	7 (27)	4 (18)	4 (17)
Severe problems	1 (4)	2 (8)	2 (9)	4 (17)
Unable to/extreme	1 (4)	1 (4)	0 (0)	0 (0)
Pain/discomfort				
No problems	10 (40)	6 (24)	10 (43)	9 (38)
Slight problems	10 (40)	4 (16)	8 (35)	6 (25)
Moderate problems	2 (8)	11 (44)	3 (13)	6 (25)
Severe problems	3 (12)	3 (12)	1 (4)	2 (8)
Unable to/extreme	0 (0)	1 (4)	1 (4)	1 (4)
Anxiety/depression				
No problems	18 (72)	12 (46)	13 (57)	14 (58)
Slight problems	5 (20)	6 (23)	8 (35)	4 (17)
Moderate problems	1 (4)	5 (19)	2 (9)	3 (13)
Severe problems	1 (4)	3 (12)	0 (0)	2 (8)
Unable to/extreme	0 (0)	0 (0)	0 (0)	1 (4)

N=25 in the intervention arm at 3 months; 25–26 in the control arm at 3 months; 22–23 in the intervention arm at 6 months and 24 in the control arm at 6 months.

Thirty of the 38 participants (79%; 95% CI 63% to 90%) in the intervention arm completed the full PACE-D questionnaire and another patient completed only the first of the six parts of the instrument. The median (IQR; range) time taken to complete the PACE-D was 7.1 (4.2–10.5; 2.1–36.3) minutes. Of the intervention participants who responded to questions on how they experienced the PACE-D, 90% ((19/21); 95% CI 70% to 99%) found it to be useful; 84% ((16/19); 95% CI 60% to 97%) found it convenient and easy to use; 81% ((17/21); 95% CI 58% to 95%) thought it helped them think about issues related to their care; 81% ((17/21); 95% CI 58% to 95%) thought it helped them think about their experience of diabetes; 81% ((17/21); 95% CI 58% to 95%) said that it did not increase any stress associated with their appointment and 45% ((9/20); 95% CI 23% to 68%) said that it did change the way the doctor dealt with them. When comparing the length of the consultation, there was little evidence of a difference between the intervention and control arms (mean (SD): 20.2 (12.1) versus 21.8 (8.4) minutes; mean difference: -1.6; 95% CI −8.3 to 5.1; p=0.64).

## Discussion

This feasibility study experienced difficulties in recruiting and following up trial participants. Therefore, despite participant reports that the intervention (PACE-D) was acceptable to a majority of patients and was not associated with an increase in the length of the clinic consultation, it would not be feasible to run a definitive randomised controlled trial using the current protocol without further development.

This study provides insights into the challenges of conducting a complex trial in everyday clinical practice as shown by the fact that we were able to recruit less than one in five eligible patients into this study. As healthcare team members are often faced with severe time pressures at their work, a key to successful implementation of a new care delivery intervention is to fit in the intervention with existing workflows and cause minimal disruptions on current workflow practices.^26^ In our study, the process of recruitment, the logistics of the intervention and the extent of data collection in terms of outcome measures appeared too complex, and seemed to have put an extra demand on patients and healthcare professionals impacting on recruitment and on data completeness. Data on HbA1c, the intended primary outcome of the definitive trial, were obtainable for only 86% of participants at baseline, 45% at the 3 month follow-up and 73% at the 6 month follow-up; this may reflect the frequency of HbA1c measurements that patients with diabetes undergo in the routine clinical practice. If such data are to be obtained in a definitive trial, their collection needs to be built into the research or the timings for inclusion of routine measurement of this outcome need to be more flexibly and realistically defined. Data accrual for the questionnaire-based secondary outcome measures was, however, also similarly inadequate. For a future definitive randomised controlled trial, major modifications to the protocol will be required so that these processes are made much simpler and more seamless to improve recruitment and data collection.

The majority of patients who completed the PACE-D found the intervention acceptable, easy to use and helped them think about issues related to their care; nearly half of them reported that it changed the way the doctor dealt with them. We recruited people with known diabetes, many of whom had attended the clinics before and some of whom know the clinic staff quite well. Our findings suggest that even for this group, for whom it is more likely that their major questions had already been addressed, the PACE-D tool is potentially useful. We would speculate that newly diagnosed diabetes patients may benefit to a still greater degree.

Our findings are consistent with those of a previous study which showed that an online communication tool based on the PACE system is highly acceptable to patients with cancer.^27^ The time and input from healthcare assistants that patients required for completing the PACE-D tool in our study was highly variable, suggesting some patients may be more comfortable using the intervention than others. There was a general perception among consultants that PACE-D would increase the consultation time. However, the length of the consultation was found to be similar in the intervention and control arms of the study. This study also showed that, with appropriate training, this intervention could be delivered by healthcare assistants, and the scarce resources of diabetes specialist nurses are unnecessary for its delivery.

A strength of this study is the participation of PPI representatives (16 altogether) at all stages of the study, including proposing the research question, study design, intervention development, training of healthcare assistants, analysis of qualitative data, steering committee membership and preparation of the protocol paper.^13^ This study shows how PPI representatives can make a valuable contribution to study design and data analysis, and how PPI representatives and healthcare professionals can work together in the design and implementation of a clinic trial. This will be reported more fully in another paper.

The study examined several outcomes. Although the Patient Report of Communication measure is designed to capture the impact of the PACE-D, it is questionable whether the other patient-centred outcomes and glycaemic control do. Lack of a theoretical basis for an intervention effect also applies to other potential clinical outcomes not measured here that are relevant to diabetes control, such as lipid levels and blood pressure. The available literature on the pathway between communication and health outcomes^28^ ^29^ may provide a framework for choosing the appropriate measures for evaluating the PACE-D intervention in a definitive study.

## Conclusion

The study suggests that the modified version of the PACE tool was acceptable to patients and helped them to articulate their agendas before an outpatient clinic appointment. It also suggests that this intervention, despite the initial apprehensions of clinical staff, did not lead to longer consultations. Although the study demonstrated that the randomised controlled trial of a web-based preconsultation intervention tool as set out in our original protocol is not feasible, it provides insights into the role of agenda forms in medical consultations. It also provides insights into the difficulties of conducting health services research in clinical settings and the need to have proportionate recruitment procedures for non-clinical interventions. Finally it, adds to our learning about the involvement of PPI representatives in trials of complex interventions.
