# Supplementary material for: A preconsultation web-based tool to generate an agenda for discussion in diabetes outpatient clinics to improve patient outcomes (DIAT): a feasibility study
Source: BMJ Open. 2017 Mar 7;7(3):e013519. doi: 10.1136/bmjopen-2016-013519 (PMC5353257; doi:10.1136/bmjopen-2016-013519)
Supplement: supplementary material [file bmjopen-2016-013519supp.pdf]

Agenda Form for Participant 2

A particular concern?

Important?	Questions or concerns	Further information
☑	I have come to the clinic with a particular concern that I would like to discuss with the Consultant	being tired, no energy, sleeping for hours, waking with heavy sweats
	I am concerned by genital itching or regular episodes of thrush	Yes

A particular concern about diabetes?

Important?	Questions or concerns	Further information
	I have a particular question or concern about my diabetes	nothing seems to be working

A concern about medication?

Important?	Questions or concerns	Further information
	Am I on the right dose?	Yes

A concern about tests or investigations?

Important?	Questions or concerns	Further information
	I have a particular question or concern about a diabetes test or examination	am i testing myself often enou

A concern about managing diabetes?

Important?	Questions or concerns	Further information
	I have a particular question or concern about ways of managing my diabetes	when will i get my foot appoin
☑	When will I see results?	Yes

A question about following treatments?

Important?	Questions or concerns	Further information
☑	I have my own question about following treatments	i'm not sure that i get the ti

---

Checking Your Understanding

- Ask the doctor to repeat or clarify information that is unclear.
- Repeat aloud what the doctor has just said.
- Summarize your understanding of what the doctor has just said.

CONFIDENTIAL

OFFLINE PACE-D INTERVENTION

DIAT

Diabetes Intervention for Agenda Trial

**Participant Study Number:**	☐☐☐☐☐
**Participant Initials:**	☐☐☐

Sponsor: Royal Devon & Exeter Hospitals NHS Foundation Trust

Chief Investigator: Professor Nicky Britten

ISRCTN: ISCRTN75070242

REC Reference: 13/NW/0123

Version: Final 1.0_27.06.2013

PenCTU/2012/001

PAGE 1

Diabetes Intervention for Agenda Trial

Thank you for agreeing to take part in this study.

A Healthcare Assistant (HCA) will support you in completing this questionnaire. It is aimed at helping you to identify important areas for discussion in the consultation with your consultant. **This may enable you to play a more active role in the consultation and subsequently make you more confident and successful in managing your diabetes.**

On each page you will be asked to tick some boxes, or write in your answers. There are ‘suggestion pages’ that you might wish to view, and before you finish you should take the opportunity to review and amend any answers. It should take you 20-30 minutes to complete.

Take your time and turn the page when you are ready.

PAGE 2

To produce your 'Agenda Form'

This questionnaire has four parts. Two parts involve answering questions and two parts consist of advice and tips:

- **Present detailed information about how you feel;**
- **Ask questions which are of interest to you;**
- **Check your understanding;**
- **Express any concerns.**

The aim of the questionnaire is for YOU to identify YOUR agenda for the consultation.

After you have completed the questionnaire, you will proceed to the clinical consultation, where the completed questionnaire (or 'agenda form') will act as an agenda for the consultation.

PAGE 3

Present detailed information about how you feel

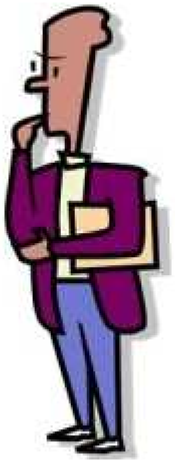

It is especially important for you to think about how you are feeling, your issues and questions.

Have you come to the clinic with a particular concern that you would like to discuss with the consultant?

Yes ☐ No ☐

If yes, please give details:

On the following page there are some suggestions of issues that you may like to discuss.

Present detailed information

It is especially important for you to think about how you are feeling, what matters to you, and any questions. Below is a list of issues that some other patients have raised. Are you concerned by? (Tick as many as apply)

Passing urine more often than usual, especially at night	☐
I feel low	☐
Slow healing of cuts and wounds	☐
Extreme tiredness	☐
I feel anxious	☐
Unexplained weight loss	☐
Skin infections	☐
I feel sad	☐
Genital itching or regular episodes of thrush	☐
Increased thirst	☐
I feel depressed	☐
Blurred vision	☐
Cramps	☐
I feel worried	☐
Sexual problems	☐
Constipation	☐
**Or: I am not concerned by any of these**	☐

PAGE 5

Asking questions

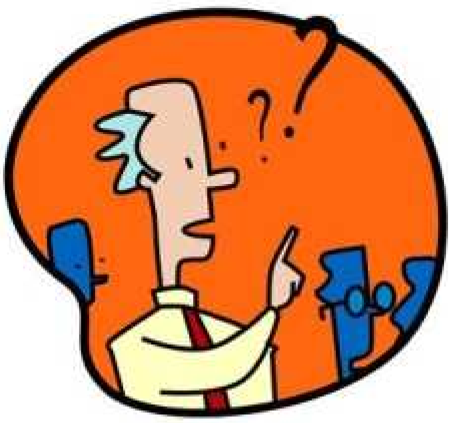

Thinking about questions to ask the consultant before the appointment will help you to decide what information is important for you to get during your consultation. This section contains sample questions about topics that may be of interest to you.

If you are stuck there will be some sample questions.

Concerns about living with diabetes?

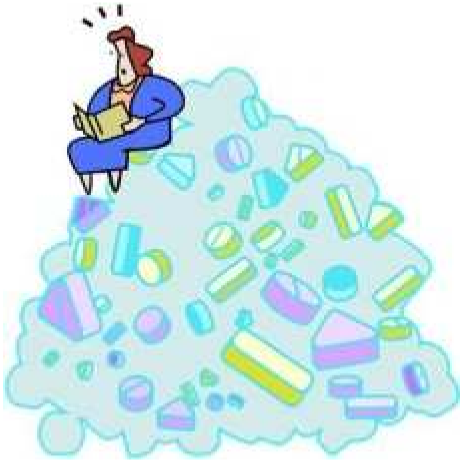

Do you have a particular question or concern about living with diabetes?

Yes ☐ No ☐

If yes, please give details:

On the following page there are some suggestions of questions that you may like to discuss.

Sample questions about living with diabetes

What is diabetes?	☐
Why do I have diabetes?	☐
How serious is diabetes?	☐
Can I expect to improve, remain the same, or get worse?	☐
Will diabetes affect my normal activities? If so, how?	☐
How long will I have to follow the treatment for diabetes?	☐
How will I know if the condition is improving or getting worse?	☐
Where can I find more information about diabetes?	☐
Where may I contact support groups for diabetes?	☐
**Or: Is there another matter or question about your diabetes that you would like to discuss?**	☐

If yes, please give details:

Or: I am not concerned by any of these ☐

Concerns about your medications?

It is important for you to tell your consultant what medications you are taking, including those prescribed by other doctors and non-prescription vitamins, minerals and herbs.

Do not assume that you consultant will remember all of the medications you are taking, even if he or she prescribed them.

Are there any issues about a medicine that you would like to discuss?

Yes ☐ No ☐

If yes, please give details:

On the following page there are some suggestions of questions that you may like to discuss.

Sample questions about medications

Why am I taking this medicine?	☐
What does this medicine do?	☐
Does this medicine have any side effects?	☐
Will it interact with other medicines, vitamins or herbs I am taking?	☐
How will I recognize negative reactions or effects? What should I do if I experience any of these?	☐
Are there other choices?	☐
Is there a different medicine?	☐
Are there ways to treat my diabetes without medication?	☐
Am I on the right dose?	☐
What will happen if I don't take the medicine?	☐
How much better will I feel after taking the medicine?	☐
How long until I feel better taking this medicine?	☐
How should I take it? How often should I take it?	☐
How long will I need to take it?	☐
What should I do if I miss a dose?	☐
**Or: Is there another matter or question about medications that you would like to discuss?**	☐

If yes, please give details:

Or: I am not concerned by any of these ☐

Concerns about tests or investigations?

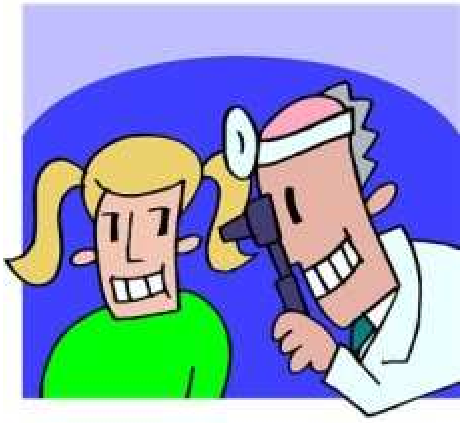

Do you have a particular question or issue about a diabetes test or investigation?

Yes ☐ No ☐

If yes, please give details:

On the following page there are some suggestions of questions that you may like to discuss.

Sample questions about tests or investigations

Am I testing myself enough?	☐
How will I know if I need a particular test?	☐
Who does the test or procedure?	☐
Will it hurt?	☐
How is it done and what steps are involved?	☐
How long will I have to wait?	☐
Where is it done?	☐
How long will it take?	☐
Do I do this myself?	☐
Who books the test/procedure?	☐
How will I understand the results?	☐
Who will help me understand what that means?	☐
**Or: Is there another matter or question about a test or examination that you would like to discuss?**	☐

If yes, please give details:

**Or: I am not concerned by any of these**	☐

PAGE 12

Concerns about other ways of managing your diabetes?

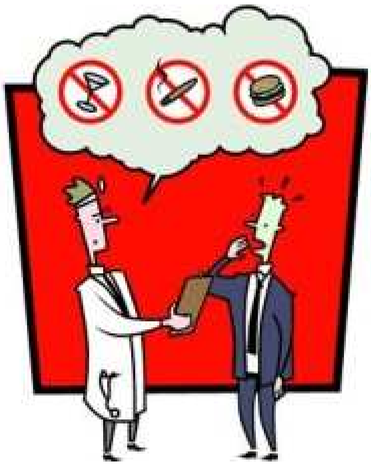

Do you have a particular question or issue about ways of managing your diabetes?

Yes ☐ No ☐

If yes, please give details:

On the following page there are some suggestions of questions that you may like to discuss.

Sample questions about other ways of managing your diabetes

Why should I manage my diabetes? ☐

What happens if I don't? ☐

How should I manage my diabetes? ☐

How long will I need to do it? ☐

What are the advantages and disadvantages of doing it? ☐

When will I see results? ☐

How will I recognize negative reactions or effects? ☐

Are there risks? ☐

Are there other choices? ☐

Or: Is there another matter or question about other ways of managing diabetes that you would like to discuss? ☐

If yes, please give details:

Or: I am not concerned by any of these ☐

Check your understanding (Tips)

It is a good idea to check your understanding of information the consultant gives you, such as directions for taking a medication. There are three ways you can do this:

- **Ask the consultant to repeat or clarify information that is unclear.**
- **Repeat aloud what the consultant has just said.**
- **Summarize your understanding of what the consultant has just said.**

Sometimes patients are shy about checking their understanding, but doing so is important. It shows the consultant that you are concerned about getting complete and correct information. You are more likely to remember the information, especially if you summarize what the consultant has told you in your own words. Ask the consultant to spell medical terms, or write down information for you.

PAGE 15

Expressing your concerns about the recommended treatment (Tips)

This is your chance to tell the consultant things you find hard about following recommended treatments.

For example don't hesitate to explain exactly why you struggle to take your medication as prescribed.

Express your willingness to work openly with the consultant. Together you will be able to devise a treatment plan which suits you better and may improve your health.

Do you have a comment about your treatment?

Yes ☐ No ☐

If yes, please give details:

PAGE 16

Thank you for completing this questionnaire

Please now review your answers and make any required changes. You may have many questions or concerns you want to discuss with the consultant but there may not be enough time to go through all of them today. To ensure the points most important to you are discussed please write your 3 most important questions or concerns in the space provided below. Let the nurse know when you have finished or if you have any problems.

A particular concern?

A particular concern about diabetes?

A concern about medication?

A concern about tests or investigations?

A concern about managing diabetes?

A question about following treatments

Check your understanding

- Ask the consultant to repeat or clarify information that is unclear.
- Repeat aloud what the consultant has just said.
- Summarize your understanding of what the consultant has just said.

Screen 1

Diabetes Intervention for Agenda Trial
Thank you for agreeing to take part in this study.

A Healthcare Assistant (HCA) will support you in completing this electronic questionnaire. It is aimed at helping you to identify important areas for discussion in the consultation with your consultant. This may enable you to play a more active role in the consultation and subsequently make you more confident and successful in managing your diabetes.

On each screen you will be asked to press some buttons, or type in your answers. There are 'suggestion screens' that you might wish to view, and before you finish you will have the opportunity to amend the form before you print it off. It should take you 20-30 minutes to complete.

Take your time and press the continue button when you are ready.

Screen 2

To produce your 'Agenda Form'

This electronic questionnaire has four parts. Two parts involve submitting questions and two parts consist of advice and tips:

Present detailed information about how you feel;

Ask questions which are of interest to you;

Check your understanding;

Express any concerns.

The aim of the electronic questionnaire is for YOU to identify YOUR agenda for the consultation.

After you have completed the questionnaire, you will proceed to the clinical consultation, where the completed questionnaire (or 'agenda form') will act as an agenda for the consultation.

Screen 3

Present detailed information about how you feel

It is especially important for you to think about how you are feeling, your issues and questions.

Have you come to the clinic with a particular concern that you would like to discuss with the consultant?

Yes/No

Screen 4

Present detailed information
It is especially important for you to think about how you are feeling, what matters to you, and any questions. Below is a list of issues that some other patients have raised. Are you concerned by? (Tick as many as apply)

Passing urine more often than usual, especially at night

I feel low

Slow healing of cuts and wounds

Extreme tiredness

I feel anxious

Unexplained weight loss

Skin infections

I feel sad

Genital itching or regular episodes of thrush

Increased thirst

I feel depressed

Blurred vision

Cramps

I feel worried

Sexual problems

Constipation

Or: Is there another matter or question about an issue that you would like to discuss?

Or: I am not concerned by any of these

Screen 5

Asking questions
Thinking about questions to ask the consultant before the appointment will help you to decide what information is important for you to get during your consultation. This section contains sample questions about topics that may be of interest to you.

If you are stuck there will be some sample questions.

Screen 6

Concerns about living with diabetes?
Do you have a particular question or concern about living with diabetes?

Yes/No

Screen 7

Sample question about living with diabetes

What is diabetes?

Why do I have diabetes?

How serious is diabetes?

Can I expect to improve, remain the same, or get worse?

Will diabetes affect my normal activities? If so, how?

How long will I have to follow the treatment for diabetes?

How will I know if the condition is improving or getting worse?

Where can I find more information about diabetes?

Where may I contact support groups for diabetes?

Or: Is there another matter or question about your diabetes that you would like to discuss?

Or: I am not concerned by any of these

Screen 8

Concerns about your medications?
It is important for you to tell your consultant what medications you are taking, including those prescribed by other doctors and non-prescription vitamins, minerals and herbs.

Do not assume that your consultant will remember all of the medications you are taking, even if he or she prescribed them
Are there any issues about a medicine that you would like to discuss?

Yes/No

Screen 9

Sample question about medications

Why am I taking this medicine?

What does this medicine do?

Does this medicine have any side effects?

Will it interact with other medicines, vitamins or herbs I am taking?

How will I recognize negative reactions or effects? What should I do if I experience any of these?

Are there other choices?

Is there a different medicine?

Are there ways to treat my diabetes without medication?

Am I on the right dose?

What will happen if I don’t take the medicine?

How much better will I feel after taking the medicine?

How long until I feel better taking this medicine?

How should I take it? How often should I take it?

How long will I need to take it?

What should I do if I miss a dose?

Or: Is there another matter or question about medications that you would like to discuss?

Or: I am not concerned by any of these

Screen 10

Concerns about tests or investigations?

Do you have a particular question or issue about a diabetes test orinvestigation?

Yes/No

Screen 11

Sample questions about tests or investigations

Am I testing myself enough?

How will I know if I need a particular test?

Who does the test or procedure?

Will it hurt?

How is it done and what steps are involved?

How long will I have to wait?

Where is it done?

How long will it take?

Do I do this myself?

Who books the test/procedure?

How will I understand the results?

Who will help me understand what that means?

Or: Is there another matter or question about a test or examination that you would like to discuss?

Or: I am not concerned by any of these

Screen 12

Concerns about other ways of managing your diabetes?

Do you have a particular question or concern about ways of managing your diabetes?

Yes/No

Screen 13

Sample questions about other ways of managing your diabetes

Why should I manage my diabetes?

What happens if I don't?

How should I manage my diabetes?

How long will I need to do it?

What are the advantages and disadvantages of doing it?

When will I see results?

How will I recognize negative reactions or effects?

Are there risks?

Are there other choices?

Or: Is there another matter or question about other ways of managing diabetes?

Or: I am not concerned by any of these

Screen 14

Check your understanding (Tips)
It is a good idea to check your understanding of information the consultant gives you, such as directions for taking a medication.
There are three ways you can do this:

Ask the consultant to repeat or clarify information that is unclear.

Repeat aloud what the consultant has just said.

Summarize your understanding of what the consultant has just said.

Sometimes patients are shy about checking their understanding, but doing so is important. It shows the consultant that you are concerned about getting complete and correct information. You are more likely to remember the information, especially if you summarize what the consultant has told you in your own words. Ask the consultant to spell medical terms, or write down information for you.

Screen 15

Expressing your concerns about the recommended treatment (Tips)

This is your chance to tell the consultant things you find hard about following recommended treatments.

: For example don't hesitate to explain exactly why you struggle to take your medication as prescribed.

Express your willingness to work openly with the consultant. Together you will be able to devise a treatment plan which suits you better and may improve your health.

Do you have a comment about your treatment?

Yes/No

Screen 16

Thank you for completing this questionnaire!

Below is a preview of your Agenda Form. If you wish to make changes you can do so by clicking Go back to previous screen.
You may have many questions or concerns you want to discuss with the consultant but there may not be enough time to go through all of them today. To ensure the points most important to you are discussed please mark your 3 most important questions or concerns by clicking the tick boxes in the tables below.
When you are happy you can print your Agenda Form. Let the nurse know when you have finished or if you have any problems.
